# Cessation of contact lenses prior to corneal tomography for keratoconus monitoring: results from a clinician survey

**DOI:** 10.1038/s41433-024-03352-2

**Published:** 2024-10-11

**Authors:** Colm McAlinden, David Lockington

**Affiliations:** 1 Department of Ophthalmology, Princess of Wales Hospital, Wales, UK; 2 Cardiff University, Cardiff, Wales UK; 3 Eye & ENT Hospital of Fudan University, Shanghai, China; 4 Wenzhou Medical University, Wenzhou, China; 5 Tennent Institute of Ophthalmology, Gartnavel General Hospital, Glasgow, UK

**Keywords:** Eye diseases, Outcomes research

Corneal tomography is essential in diagnosing and monitoring corneal ectasias, including keratoconus. Monitoring may be necessary to determine whether the disease is progressing and if corneal cross-linking is indicated, or to track progression following corneal cross-linking. It is important for tomographers to provide precise (repeatable and reproducible) measurements to detect progression [1], although achieving this precision is more challenging in eyes with keratoconus [2]. Visual improvement in eyes keratoconus is typically managed with hard contact lenses. Contact lenses may induce tomographic changes to the corneal shape. This can interfere with our ability to accurately distinguish between pathological changes due to disease progression and temporary physiological changes caused by contact lens usage.

Corneal warpage was first reported by Hartstein et al. in 1965 in a case series involving 12 patients with increased astigmatism from PMMA contact lenses [3]. PMMA lenses impede the delivery of oxygen to the cornea and the removal of carbon dioxide, causing acidosis. Hypoxia and acidosis lead to corneal endothelial dysfunction (polymegathism) and subsequent corneal oedema. PMMA lenses are no longer used, but modern rigid gas permeable (RGP) and soft contact lenses still induce hypoxia to a lesser extent [4]. Furthermore, contact lenses can cause various corneal changes, including warpage/corneal exhaustion syndrome, epitheliopathy/dry eye disease, contact lens binding/moulding/wrinkling, dimple veil, and staining at 3 o’clock and 9 o’clock positions, as well as dellen. These changes can significantly affect corneal tomographic parameters [5]. Additional influencing factors include lens type (rigid, soft, hybrid), fit (corneal, limbal, scleral), lens material, wear time, duration of wear, and fit type (flat, steep, orthokeratology).

Due to the lack of published literature, it can be difficult for practicing ophthalmologists to advise patients appropriately regarding their lens wear prior to their clinic visit. Ideally, patients would discontinue contact lens wear until all *temporary* corneal changes induced by the lenses have resolved, undergo tomography scanning, and then resume wearing their lenses. However, the duration required for these changes to resolve may vary among patients due to the factors mentioned above. Additionally, some patients heavily rely on contact lenses, and ceasing lens wear for extended periods may not be feasible or acceptable to them.

In the absence of any evidence-based guidelines, we surveyed members of the United Kingdom & Ireland Society of Cataract & Refractive Surgeons (UKISCRS) and the British Society for Refractive Surgery (BSRS). Additionally, we invited attendees of the UKISCRS Cataract and Cornea Day held in Bristol, UK in February 2024 to complete the survey. A survey (designed using Google Forms) was circulated, asking the following questions:

1. When assessing a patient with keratoconus for disease progression, how long do you request the patient ceases soft contact lenses prior to the tomography scan?
2. When assessing a patient with keratoconus for disease progression, how long do you request the patient ceases rigid contact lenses prior to the tomography scan?

Response options for both questions were: 4 weeks, 3 weeks, 2 weeks, 1 week, 48 h, 24 h, 1 h, and immediately prior to the scan.

We received 45 responses: 26 from members of UKISCRS, 4 from members of BSRS, 10 from members of both, and 6 from neither UKISCRS nor BSRS members. Among the respondents, 40% were aged between 46 and 55 years, 22% between 56 and 65 years, and 18% between 36 and 45 years. Regarding grade, 86% were Consultant Ophthalmologists, 7% were Optometrists, 5% were SAS doctors, and 2% were specialty trainees in Ophthalmology.

**Fig. 1:**
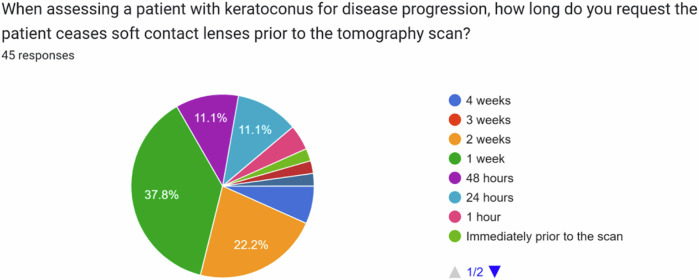
Time period requested for patients to stop soft contact lens wear prior to tomography when monitoring for keratoconus progression.

**Fig. 2:**
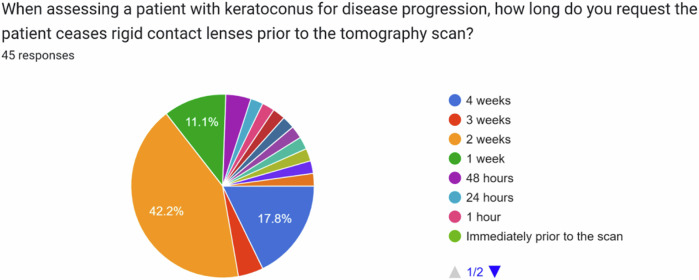
Time period requested for patients to stop rigid contact lens wear prior to tomography when monitoring for keratoconus progression.

The most popular time-period to ask patients with keratoconus to stop wearing soft contact lenses prior to tomography for monitoring disease progression was 1 week (see Fig. 1). For patients with keratoconus using rigid contact lenses, it was 2 weeks (see Fig. 2).

It was notable that a small number of respondents indicated they ask the patient to remove their lenses immediately prior to the scan or to apply the same time-period for each clinic visit. These opinions were discussed at the UKISCRS Cataract and Cornea Day and seemed to be based on the premise that any temporary corneal changes would be consistent at each visit for that individual patient using a particular type of contact lens. However, this assumption may not hold true and is not supported in the currently published literature.

We believe both patients and clinicians would benefit from a consensus leading to modern guidelines regarding the cessation of contact lenses prior to corneal tomography in keratoconus evaluation, with further investigation required to provide an evidence-base for current behaviours.
